# A Study on the Mortality and Complication Rates Following Percutaneously Adjustable Pulmonary Artery Banding

**DOI:** 10.15171/jcvtr.2014.021

**Published:** 2014-12-30

**Authors:** Ali Changizi, Alireza Yaghoubi, Mitra Azarasa, Shamsi Ghaffari, Hossein Montazerghaem

**Affiliations:** ^1^Bou ali Sina Hospital, Qazvin University of Medical sciences, Qazvin, Iran; ^2^Cardiovascular Research Center, Tabriz University of Medical Sciences, Tabriz, Iran; ^3^Cardiovascular Research Center, Hormozgan University of Medical Sciences, Bandar Abbas, Iran

**Keywords:** Pulmonary Artery, Banding, Percutaneous

## Abstract

***Introduction:*** Pulmonary artery (PA) banding is a procedure associated with high morbidity and mortality rates. It however can effectively palliate several forms of congenital heart lesions with increased pulmonary flow. Occasionally, to obtain an optimal degree of banding following operation, readjustment of the band is inevitable. We describe the technique of adjustable PA banding to prevent this problem.

***Methods:*** From June 2007 to 2008, 21 patients with congenital cardiac abnormalities including Single ventricle (1), transposition of great arteries (TGA) (4) and ventricular septal defect (VSD) (16) were operated via percutaneously adjustable PA banding in Madani Hospital (Tabriz, Iran).

***Results:*** The mean age and the mean weight of the patients were 12±.8 months and 61±.7 kg respectively. Seventeen (81%) patients survived the operation. Cause of death was heart failure in 2 (9.5%) patients, and arrhythmia in 2 (9.5%) patients. Later, patients were followed up for 6 months. Satisfactory band gradient was achieved between 48 and 240 hours. Mean PA gradient before and 1 and 6 months after adjusting was (55.3±7.1 mmHg), (54.7±5.1 mmHg), and (53.2±5.4 mmHg) respectively. In the follow up period, there were 2 deaths, one caused by aspiration pneumonia and one caused by poor mixing. Postoperative complications were observed in 28.5% of the cases including cardiac (10%), pulmonary (pneumothorax, pneumonia) (10%) and infectious complications (9%).

***Conclusion:*** The technique of percutaneously adjustable PA banding is simple and inexpensive and allows easy band adjustments without the need for multiple reoperations. Moreover, our assessment reveals that created gradient is constant and did not decrease with time.

## Introduction

Pulmonary artery (PA) banding is a palliative surgery associated with high morbidity and mortality rates. Although primary repair of most congenital cardiac anomalies is optimal, PA banding is still used as a palliative technique in certain groups of patients. PA banding is associated with high mortality due to the sudden increase in right ventricular pressure load.^1^ For instance, in transposition of great arteries (TGA) due to intolerance by left ventricular systolic pressure, PA banding is necessary.^2,3^ The problem on setting a proper extent of banding leads to tight and in some cases loss banding is required. In either case, associated complications are relatively high and subsequent surgery eventually needed.

For tightening the banding, no definite recommendation exists. It has been proven that the set pressure during surgery alone is not effective in assessing the adequacy of banding. This is probably due to gradient created while the patient receiving a high percentage of oxygen and being paralyzed and anesthetized by medications.

In critically ill patients, precious banding is not only needed during operation but also after surgery. The change of hemodynamic and respiratory conditions in patients after surgery may change gradient created during the operation. We present a case series of 21 patients with different congenital cardiac diseases including Single ventricle, TGA and ventricular septal defect (VSD) who operated using percutaneously adjustable PA banding in a referral university hospital.

## Materials and methods

A standard anesthesia technique with endotracheal intubation and intravenous medications was used. A small cannula in the radial artery for arterial pressure monitoring was placed and two peripheral veins for intravenous access were cannulated. Explaining the advantages and disadvantages of surgery, written informed consents were obtained from all patients’ parents. Median sternotomy incision was preferred. Thymus was excised partially following sternotomy. Then, pericardium was opened for limited access only to large vessels. Aortic and pulmonary arteries were separated. Ductus arteriosus was isolated in all patients and closed with clips. Ethibond 2 was passed around pulmonary arteries using forceps.

Gore-Tex strip connecting the adventitia of PA with 6-0 prolene prevented from ethibond displacement. Clips (ethicon-200) was placed on ethibond, right at the exit of cortex strip. After placing pacemaker and pericardial closure, ethibond was brought out onto the outside edge of sternum and after passing through the cortex it was fixed in subcutaneous and tied together. Later, sternum was closed.

After extubation, tightening of banding was performed while the patient breathing. Tightening of bandings was achieved within 3 to 6 stages, in the 1 to 3 day intervals, and in each stage not more than 30 mmHg gradient was added.

In two patients, desired gradient for banding was 60 mmHg with minimum O_2_ saturation of 85% in arterial blood. In patients with single ventricle, banding was performed as tight as possible, however saturation did not decrease to less than 75 mmHg. In case with hemodynamic disturbance, blood desaturation and bradycardia during tightening of banding, the last clips were removed. After creating the desired gradient, the band was placed under the skin.

In one and six months follow up after discharge, the patients were evaluated with echocardiography and O_2_ saturation and gradient were measured.

## Results

Twenty-one patients (12 males and 9 females) studied from September 2009 to 2010 were operated with adjustable PA banding in Madani Hospital, Tabriz, Iran. All patients were evaluated by pediatric cardiologist. Mean age of the patients was 12.8 months (2-35 months). Mean weight of the patients was 6.9 kg (3-10 kg). There were 16 cases of ventricular septal defect, 1 case of single ventricular and 4 cases of TGA. Various forms of VSD defects included subaortic, muscular, and atrioventricular types. Mean time of mechanical ventilation in patients undergoing surgery was 40.2 hours (3-240 hours). Banding of patients was entirely performed in the ICU. Mean time of ICU stay was 100.5±44.6 hours. Means of the gradient after the final tightening in ICU were (54.7±5.1 mmHg) and (53.2±5.4 mmHg) one and six months, respectively. After one and 6 months follow up, these measurements were 54.4±5.1 and 253.2±5.4 mmHg, respectively.

Mean required time for creating appropriate gradient was four days and subsequently the band was moved under the skin.

In one patient after one month, echocardiographic evaluation showed pulmonary artery gradient reduction from 55 mmHg to less than 30 mmHg. Consequently, the patient underwent re-banding through the skin successfully. The mean gradient of pulmonary artery in early post-operative period and at 1 and 6 months after surgery based on the Kolmogorov-Smirnov test was evaluated. In fact, the distribution and dispersion gradient was consistent with normal distribution and dispersion. Four deaths were observed; 2 cases due to arrhythmia and 2 cases due to heart failure. Survival rate at 6 months following surgery was 81%.

In one case, due to not putting clips on the ethibond under the skin, we had to re-operate. Complications were observed in six patients including cardiac complications: arrhythmia (1), and bradycardia (1), pulmonary complications: pneumothorax (1), pneumonia (1), and skin complication; superficial (above the deep fascia) and deep (below deep fascia) infection (2 cases).

## Discussion

From 1992 to 2001, more than 16 methods of PA banding were introduced. Most of these methods were expensive and special equipment was required.^4^ The presented method in this study is based on an article published in 2006 by Choudhary et al.^1^ This method is inexpensive and safe and does not require special equipment.^3^ In this method, the tightening of banding is gradual. Corno and colleagues in 2007 introduced Flow watch as a safe method for adjustable pulmonary artery banding stating that it seems to be better than traditional methods due to the following reasons^5^:

1. It does not require reoperation.
2. Control of flow rate is simple and mechanical ventilation duration and ICU and hospital stay are significantly reduced.
3. Costs are significantly reduced due to reduced ventilation time in the hospital and ICU.

Choudhary et al.^1^ in the resumption of their previously published article in 2006, concluded that the mortality rate & reoperation was less in the new method. However, no difference in ventilation time and duration of hospitalization in the two groups was observed. Results from this study showed that pulmonary artery banding through the skin is a reliable method to create the necessary gradient in patients with anomalies of heart who require banding. Following-up the patients with echocardiography showed that this gradient remains stable. In Choudhary et al.^1^ study, mortality rate in this method was less than classical methods. Additionally, complications were minimal and even could be minimized with increased medical team experience. In present case series study, our assessment reveals that created gradient is constant not decreasing in course of time. This procedure would allow us to prevent reoperation and decrease complications.

## Ethical issues

The study was approval by the Local Ethics Committee.

## Competing interests

Authors declare no conflict of interests in this study.
